# Timing of arrival from spring migration is associated with flight performance in the migratory barn swallow

**DOI:** 10.1007/s00265-012-1429-x

**Published:** 2012-10-16

**Authors:** Piotr Matyjasiak

**Affiliations:** 1 Centre for Ecological Research PAS, Konopnickiej 1, Dziekanów Leśny, 05-092 Łomianki, Poland; 2 Museum and Institute of Zoology PAS, Wilcza 64, 00-679 Warsaw, Poland

**Keywords:** Arrival timing, Condition dependence, Flight performance, *Hirundo rustica*, Migration

## Abstract

Timing of arrival at the breeding grounds by migratory birds affects their mating success and access to superior resources, thus being a major factor associated with fitness. Much empirical work has been devoted to investigate the condition dependence of arrival sequence of migrants and characteristics of individuals that influence arrival time from migration. Surprisingly, there are no studies examining the relationship between flight performance of individual birds and their arrival time. I investigated the relative importance of direct effects of short-term flight performance, age, body condition and the degree of sexual ornamentation (tail length) on timing of spring arrival in the barn swallow (*Hirundo rustica*), a long-distance trans-equatorial passerine migrant. I evaluated short-term flight performance (a composite variable comprising flight manoeuvrability, velocity and acceleration) in a standardised manner using flight tunnels. Short-term flight performance was a significant and important predictor of spring arrival date. Furthermore, locomotion predicted arrival date of individual birds independently of morphological variables—the degree of sexual ornamentation (the length of the tail) and wing aspect ratio and body condition. I discuss the possible role short-term flight performance may have in determining migratory performance. This is the first time flight performance has been shown to be associated with timing of arrival from migration in a migratory bird.

## Introduction

In migratory birds, early arrival at the breeding area is a major determinant of fitness as it gives prior access to mates in prime condition (Møller 1994a; Lozano et al. 1996; Kleven et al. 2006) or to prime breeding habitats (Aebischer et al. 1996; Hasselquist 1998; Tryjanowski et al. 2004; Gunnarsson et al. 2005) and consequently enhances seasonal reproductive success (Bańbura 1986; Møller 1994b; Smith and Moore 2005a; Kleven et al. 2006; Cooper et al. 2011). Early arrival advantages apply to both sexes (Smith and Moore 2005a; Alerstam et al. 2006; Canal et al. 2012), although they are greater for males, which usually arrive at the breeding grounds before females (Kokko et al. 2006; Newton 2008). Hence, migrants are assumed to be under pressure to return to breeding areas early in spring (Alerstam 2006; Newton 2008). Indeed, numerous studies have found that spring migration is faster than autumn migration (Alerstam et al. 2006; Stutchbury et al. 2009; Karlsson et al. 2012; Tøttrup et al. 2012; but see Shamoun-Baranes et al. 2003).

But long-distance migration is costly in terms of time, energy and predation (Berthold 2001), as well as there are limits to earliness because of survival costs that may arise due to exposure to adverse weather or insufficient food supply early in spring (Møller 1994a; Brown and Brown 1998, 1999a, 2000; Smith and Moore 2005b). Consequently, not all individuals in populations are able to arrive early. Several traits have been found to influence arrival time. These include the intensity of sexual displays, such as expression of sexual ornaments (Slagsvold and Lifjeld 1988; Møller 1994a) or song rate and repertoire size (Kipper et al. 2006), body condition and fat reserves (Lozano 1994; Dittmann and Becker 2003; Smith and Moore 2005b), health status and immunocompetence (Pap 2002; Møller et al. 2004; Ninni et al. 2004) and cognitive abilities (Møller 2010). It has been suggested that higher aspect ratio wings with more pointed wingtips increase migratory performance (Yong and Moore 1994; Bowlin 2007). These traits are a morphological adaptation known to be associated with migration (Winkler and Leisler 1992; Leisler and Winkler 2003; Lockwood et al. 1998) and correlated with reduced energy expenditure during long-distance flight (Rayner 1988; Norberg 1990; Bowlin and Wikelski 2008). Also age differences in migration schedules have been shown, with older and/or more experienced birds typically arriving earlier than younger, inexperienced ones (Lozano et al. 1996; Dittmann and Becker 2003; Smith and Moore 2005a; Balbontín et al. 2007).

It is widely held that spring arrival sequence is condition dependent (Newton 2008). According to this mechanism, prime quality individuals are better able to cope with costs of migration and costs of earliness (costs of advancing arrival raise faster for individuals of poor than high quality; Møller 1994b; Kokko 1999). Prime quality individuals have a lower mortality cost due to earliness than poor quality ones (Møller 1994a; but see Brown and Brown 1999a). Also between-individual variation in the ability to achieve the advantages associated with earliness has been found to determine arrival sequence (Forstmeier 2002).

Surprisingly, individual variation in flight performance has never been investigated in the context of timing of spring arrival, although this trait might be an important adaptation in time-selected migration, enabling migrants to arrive early and in good condition. For instance, higher flight performance may directly translate into earlier arrival if the best fliers are able to increase their airspeeds more than others during the last stages of migration. Recent tracking-radar studies of nocturnal migrant passerine birds (Karlsson et al. 2012) have suggested that birds may sprint during the final approach to their breeding destination. Second, short-term flight performance (e.g. manoeuvring and acceleration performance) may indirectly influence the date of arrival through increased foraging rate. Houston (2000) has shown that foraging performance (determining deposition rate of fat reserves depleted during migration) has stronger effect on resulting migration speed than changes in the birds’ airspeed. Higher flight performance can aid in paying the costs of energetically expensive flight and in sustaining the costs of earliness, particularly in birds foraging on the wing and adopting the fly-and-forage strategy during migration (e.g. swallows, swifts and falcons; Strandberg and Alerstam 2007). This may apply particularly in the case of swallows and martins, which are the only migratory passerines that forage during flight. Third, higher flight performance may assist in achieving benefits associated with earliness, such as superior mates and resources (Husak and Fox 2008).

I investigated whether short-term flight performance correlates with spring arrival date in the barn swallow (*Hirundo rustica*), a long-distance trans-equatorial passerine migrant (Møller 1994b; Brown and Brown 1999b; Turner 2006). There is strong selection for early arrival in this species because of benefits in terms of partner quality, nest site availability and time to raise a second or a replacement clutch after failure (Bańbura 1986; Møller 1994b). Barn swallows arrive at their breeding grounds in Europe from February to May, depending on latitude of the breeding area (Turner 2006). In Poland, the earliest arrivals can be seen in late March or early April, with the majority of birds having arrived in late April or early May. Arrival date is thought to be condition dependent in this species, with older males in prime condition arriving earlier than older females in prime condition, and these earlier than young individuals of both sexes (Møller 1994b; Møller et al. 2003). Barn swallows possess long and dimorphic outermost tail feathers, which in males from European populations are thought to be condition-dependent signal of individual quality (Bańbura 1986; Møller 1994b; Saino et al. 1997a, b; but see Bro-Jørgensen et al. 2007; Aparicio and Møller 2012). Similar studies of the function of tail streamers in American populations of barn swallows have reached mixed conclusions, however (Brown and Brown 1999a; Safran and McGraw 2004; Kleven et al. 2006; Lifjeld et al. 2011). Spring arrival date is inversely related to the length of the tail in male and female barn swallows (Møller 1994b; Cuervo et al. 1996).

This study aimed at (1) clarifying whether short-term flight performance in individual barn swallows is related to their arrival date and (2) investigating the relative importance of direct effects of short-term flight performance, age, body condition and the degree of sexual ornamentation (tail length) in the context of condition-dependent timing of spring arrival. I evaluated short-term flight performance in a standardised manner with flight tunnels (Warrick 1998; Rowe et al. 2001; Bowlin and Winkler 2004; Matyjasiak et al. 2004, 2009).

## Methods

### Study site and general methods

The study was carried out in two barn swallow colonies (ca. 30 and 20 breeding pairs) located in two horse stables situated 1.5 km apart in the Łomianki commune near Warsaw (52°21′ N, 20°53′ E; elevation, 75 m), central Poland, during 2007–2008. The study area is a lowland area. The land is mostly agricultural with scattered orchards and groups of trees and is mainly used as hay meadows. Some minor crops are cereals and vegetables. There are two large oxbow lakes and a large braided river, the Vistula River.

The study population was intensively banded during a pilot study in 2006, by frequent mist netting carried out during the day and at dawn between mid-April and late August. I paid special attention to capture males that remained unpaired throughout the breeding season (mostly second-year (SY) birds that often appear in the breeding colony and pair in the next spring). According to observations of birds, all resident breeding pairs and unpaired males had been individually colour banded in both colonies. All nestlings were ringed throughout the study as well.

Date of arrival was estimated based on observations of marked birds and frequent capture sessions. Observations of arriving birds in breeding colonies started on 1 April. These were carried out three times a week between 10:00 and 14:00 using a ×20–60 scope and were continued during evenings when roosting birds were identified using a ×10 binocular and a flashlight (birds spend the night within their small territories alone, if unmated, or next to their partner, if mated; Møller 1994b).

Birds were captured two to three times a week in each farm during the arrival period between 4:00 and 10:00 from 15 April by intensive mist netting. At first capture, birds were sexed according to Svensson (1984), ringed with a numbered aluminium ring and individually marked with a combination of colour leg bands. Since breeding pairs and unmated males were intensively banded in 2006, banded birds could be classified as after second year (ASY)—birds in at least their second breeding season, while unmarked birds or birds banded as nestlings in the previous year were classified as SY—birds hatched the previous year and in their first breeding season. This approach is justified on the basis of a high breeding philopatry of this species (Møller 1994b) and on my own capture–recapture data. None of the breeding birds from 2006 to 2008 had moved to a different breeding site in a following year. I considered the date of arrival to equal the date of first observation in the colony (marked birds) or the date of first capture (unmarked birds), relative to day 1 = 1 April.

The following biometric measurements were taken: left and right flattened wing length (from the carpal joint to the tip of the longest primary feather), wingspan (the longest distance from one wing tip to the other, with the wing joints fully extended) and left and right outermost tail feather lengths, and the length of the inner tail feathers were measured to the nearest 1 mm with a ruler; head, bill, keel and left and right tarsus lengths were measured to the nearest 0.1 mm using a pair of callipers; body mass was measured to the nearest 0.5 g with a Pesola spring balance. Paired measurements were averaged to calculate mean wing, outermost tail and tarsus length. Aspect ratio, which is usually used to model the overall shape of the wing (e.g. Rayner 1988), was calculated as (wingspan)^2^/wing area. To measure wing area, wing drawings were made by making a tracing of the outlines of fully extended wings. Wing drawings were subsequently analysed in MultiScan 14.02 (Computer Scanning Systems II, Warsaw, Poland). Wing area was estimated as the area of both wings including the area of that part of the body that was included between the wings (Pennycuick 1989). After measurements and ringing, which took ca. 15 min, birds were immediately released. All measurements were taken by me.

### Measurements of short-term flight performance

Flight manoeuvrability was assessed by releasing birds through a flight maze measuring (length × width × height) 18 × 4 × 1.6 m. The maze consisted of a metal frame covered in a fine-mesh garden sunshade netting (black; shade factor, 35 %), with its long axis being oriented west–east. The west end of the maze was closed and the east end was open. The first 9-m section of the maze with the release box was free of obstacles and acted as an acceleration zone. The remaining 9-m section towards the exit acted as a test zone. It contained 16 successive panels of vertical cotton strings suspended from the roof of the maze and weighed. Both the distance between the strings within a panel and the distances between consecutive panels decreased towards the exit. The within-panel separation of the strings decreased from 70 cm at the beginning of the test zone (roughly twice the wingspan of a barn swallow) to 8 cm at the exit (roughly a quarter of the wingspan of a barn swallow). The between-panel distance decreased from 70 to 40 cm. The strings were placed so that each panel was offset from both the neighbouring panels. The birds were released (after 2 min of acclimatisation) from a box at the closed end and flew through the maze to escape from the open end. The front side of the box was opened remotely with a string and two to three moderate shakes were immediately applied to the box (with a bamboo stick) in order to motivate the birds to take off (it was enough to instantly make the birds fly). The time taken for a bird to negotiate the maze test section was used as a measure of the bird’s ability to cope with the obstacle course. A faster flight time indicate greater manoeuvrability (Rowe et al. 2001; Bro-Jørgensen et al. 2007). Time taken to negotiate the maze stringed section was measured based on video images (filming at 25 frames s^−1^; HDV camcorder SONY HDR-HC1) obtained with the use of angled mirrors positioned in line with the first and last panels of strings. A bird’s image was reflected in the first mirror as it entered the test section and the second image was reflected in the other mirror when it left the maze. Videos were analysed by viewing them frame-by-frame in Edius Pro 3 (Canopus, Reading, UK). The flight time was estimated as the number of successive video frames between the two images (accuracy of 0.04 s).

Maximum acceleration and maximum velocity were measured by releasing birds through another flight tunnel measuring 10 × 1.2 × 1.2 m. The tunnel consisted of a metal frame covered in fine-mesh netting. A small release box was centred on the closed end. It was placed bottom up on a small wooden board. The birds were placed in the box through a small hole in the bottom covered with a flap, and were released by raising the box. All birds took off within few seconds after the box had been raised. A Stalker Pro ATS radar gun (Applied Concepts Inc., Plano, TX) connected to a portable computer was mounted on a tripod at the open end of the tunnel. The radar was run with a minimum speed of 0 and a maximum of 225 kph on high range, with the auto-clear set to 0 s and the peak mode off. To minimise signal noise in the radar, the flight tunnel was positioned inside an unused building with the open end placed at the exit doors. Radar data were analysed using Stalker Pro ATS 4.5 (Applied Concepts Inc., 2002, Plano, TX), in ‘acceleration run’ mode. The program was configured to discard any data points that occurred before the swallow had been released and after it had left the tunnel. These trials resembled that of Warrick (1998) and Bowlin and Winkler (2004)—released birds were assumed to be motivated to escape from the flight tunnel as fast as possible, and flight velocity and acceleration of released birds was measured. To create velocity-versus-time and acceleration-versus-time graphs in ‘acceleration run’ mode, medium digital filter setting was used, as recommended by Stalker (Vanman and Shorten 1997). Maximum acceleration and maximum velocity were obtained from these graphs with the graph tracer.

Barn swallows were tested in the flight maze and the flight tunnel during the period of feeding first brood nestlings (aged 6–15 days). Birds were captured and tested between 6:00 and 10:00 on 4 days between 20 May and 20 June. Clear days with no wind or rain (temperatures of approximately 20 °C) were chosen for trials. First, birds were released through the flight maze (manoeuvrability trial) and recaptured in a mist-net positioned in front of the maze (the distance between the last panel of strings and the mist-net was ca. 30 cm). Immediately after the manoeuvrability trial birds were released through the flight tunnel (acceleration and velocity trial) and regained freedom.

Mean (SE) value for manoeuvrability (estimated as the time taken for a bird to negotiate the maze test section) was 1.9 s (0.04); maximum acceleration, 8.1 m s^−2^ (0.21); and maximum velocity, 7.1 m s^−1^ (0.08). Flight parameters resembled those reported in similar studies (Warrick 1998; Rowe et al. 2001), indicating reliability of my measurements of short-term flight performance. To check for the repeatability of flight measures, a sample of swallows was tested twice at an interval of a week during the pilot study in 2006. Repeatability estimates (Lessells and Boag 1987) were significant for all three flight measures (manoeuvrability—*r* = 0.46, *F*_14, 15_ = 2.71, *P* = 0.03; maximum acceleration—*r* = 0.48, *F*_16, 17_ = 2.83, *P* = 0.02; and maximum velocity—*r* = 0.86, *F*_17, 18_ = 13.01, *P* < 0.001). An important assumption of this study is that flight performance measured during the nestling feeding period is representative of arrival flight performance. I therefore checked the validity of this assumption in 2008 by testing a separate sample of birds twice—first during first capture shortly after arrival and second during nestling feeding of first brood. The repeatability estimates were high and significant for all three flight measures (manoeuvrability—*r* = 0.74, *F*_8, 9_ = 6.76, *P* = 0.005; maximum acceleration—*r* = 0.85, *F*_10, 11_ = 12.47, *P* < 0.001; and maximum velocity—*r* = 0.65, *F*_10, 11_ = 4.79, *P =* 0.008). Repeatability estimated with the birds tested in two different years of study was significant for manoeuvrability (*r* = 0.58, *F*_21, 22_ = 3.90, *P* = 0.001) and maximum velocity (*r* = 0.76, *F*_21, 22_ = 7.45, *P* < 0.001) but not for maximum acceleration (*r* = 0.26, *F*_21, 22_ = 1.69, *P =* 0.12).

### Statistical analysis

In total, 155 adult barn swallows were tested. I have excluded from the analysis 31 birds that were hovering within the test zone or at the mist net or were sitting on strings rather than flying through the test zone (no bird landed on the ground or flied to walls). The final sample thus contained 124 birds—60 in 2007 and 64 in 2008, among which 32 and 34, respectively, were males. The sample included 22 birds (10 females and 12 males) that were examined in both years. All analyses were done with the SPSS 13.0 (SPSS Inc., 2004, Chicago, IL).

Body condition index was expressed as residuals from a linear regression of body mass on the day of first capture against (tarsus length)^3^ (Tomkins et al. 2004; Balbontín et al. 2012). This index was verified by correlating residual mass and subcutaneous fat deposit score (Kaiser 1993) gauged on the first capture of a sample of birds in 2008. The correlation was significant for males (Spearman rank correlation coefficient—*r*_s_ = 0.56, *n* = 33, *P* < 0.001) and females (*r*_s_ = 0.49, *n* = 20, *P* < 0.03). Manoeuvrability, maximum acceleration and maximum velocity were inter-correlated, and I therefore used principal components to construct a new composite variable representing the birds’ short-term flight performance. Only one principal component was extracted; it was a reliable descriptor of flight performance, showing high factor loadings for all flight measures (eigenvalue = 1.55; factor loading for manoeuvrability = 0.74, maximum acceleration = 0.65 and maximum velocity = 0.76). Short-term flight performance did not differ significantly among days of flight trials (ANOVA, 2007—*F*_3, 57_ = 0.461, NS and 2008—*F*_3, 61_ = 0.485, NS), years of study (*F*_1, 123_ = 0.271, NS) or phases of the nestling feeding stage (breeding phenology was a categorical factor with three levels—nestlings aged 6–8, 9–12 and 13–15 days; 2007—*F*_2, 57_ = 1.94, NS; and 2008—*F*_2, 61_ = 1.59, NS). Tail length was an indicator of individual quality (Møller 1994b; Turner 2006). Predictor variables were not strongly inter-correlated (*r* coefficients lower than 0.3). To obtain normality and homoscedascity, arrival date, all flight measures and morphological variables were log transformed before entering them in the statistical analysis.

To test for effects of short-term flight performance, body condition, tail length and age on arrival date, I fitted linear mixed effects models with maximum likelihood estimation. Individual identity was included as a random effect to link data from the same individuals. The effects of individual predictor variables on arrival date in simple linear mixed effects models with sex, year and colony (to account for differences among the two breeding colonies) as additional predictors were first investigated. Subsequently, I developed best-fit linear mixed effects models to assess the relationship between spring arrival date as dependent variable, age, sex, year and colony as categorical factors (fixed effects), flight performance, body condition, tail length and aspect ratio as covariates. The second-order Akaike information criteria (AIC_c_), which are appropriate for small sample sizes, to select models predicting variation in the spring arrival date was used. Furthermore, all combinations of predictor variables to this end were assessed. A set of candidate models is presented in Table 1.

**Table 1:** Comparison of candidate models to investigate the relative effect of short-term flight performance, body condition, tail length and age on spring arrival date in the barn swallow

Model description	AIC	*k*	AIC_c_	Δ_*i*_ AIC_c_	*ω*_*i*_	Linear regression summary	Adjusted *R* ^2^
Best model (flight performance, body condition, tail length, age, sex and year)	64.783	9	66.362	0	0.142	*y* = 0.439*x* + 1.919	0.463
Best model − tail	65.802	8	67.055	0.693	0.101	*y* = 0.424*x* + 1.967	0.446
Best model + aspect ratio	66.643	10	68.590	2.228	0.047		
Best model − body condition	67.371	8	68.624	2.262	0.046	*y* = 0.420*x* + 1.983	0.449
Best model + colony	66.745	10	68.692	2.330	0.044		
Best model − year	68.131	8	69.383	3.021	0.031		
Best model − flight performance	68.915	8	70.168	3.806	0.021	*y* = 0.388*x* + 2.096	0.414
Best model + aspect ratio + colony	68.625	11	70.982	4.620	0.014		
Best model − sex	72.074	8	73.326	6.964	0.004		
Best model − age	108.234	8	109.486	43.124	0.000	*y* = 0.219*x* + 2.681	0.234
Null model	122.524	3	122.724	56.362	0.000		

Akaike Information Criterion (AIC), number of parameters (including the individual identity random factor and two intercepts (*k*)), second-order Akaike Information Criterion (AIC_c_), delta AIC_c_ and Akaike weights (*ω*_*i*_) are shown for each model. The last two columns show equations and variation explained (adjusted *R* ^2^) by regressing best model predicted values against observed values to compare the relative importance of short-term flight performance, body condition, tail length and age on spring arrival date. Each predictor variable was systematically excluded from the best model and only one effect was removed at a time. Flight performance is the first principal component extracted from flight manoeuvrability, maximum acceleration and maximum velocity. Condition is an index of body condition expressed as residuals from a linear regression of body mass against cubic tarsus length. Models with higher Akaike weights (*ω*_*i*_) have more empirical support. The null model included the individual identity random factor and two intercepts, without addition of predictor variables. Delta AIC_c_ was calculated as the difference between a given model and the best model

I used the following techniques to compare the relative importance of short-term flight performance, body condition, tail length and age on spring arrival date. First, cumulative predictor weights by summing the Akaike weights over all models in which the predictor variable features were calculated. Cumulative weights indicate the probability that that predictor variable is a component of the best model and hence can be used to rank various predictor variables in terms of importance (Burnham and Anderson 2002). A full-model averaging was also employed in order to obtain model-averaged parameter estimates, which indicate the effect size (Burnham and Anderson 2002). Second, I regressed the best model fitted predicted values against the observed data and repeated this procedure after removing each of the four predictor variables from the model, one at a time.

## Results

In both years, birds arrived at the study area over 8-week period, with the earliest male arriving on 11 April 2007 and 2008 and the earliest female arriving on 11 April 2007 and 21 April 2008. Overall, the first 75 % of females and the first 75 % of males arrived over a 25-day period. The first SY males arrived 10–20 days after the first ASY males, while the first SY females arrived 5–16 days after the first ASY females. Pooled across the 2 years, median arrival date for ASY males was 22 April, and for ASY females—29 April. Median arrival day for SY males was 9 May, and for SY females—10 May.

**Fig. 1:**
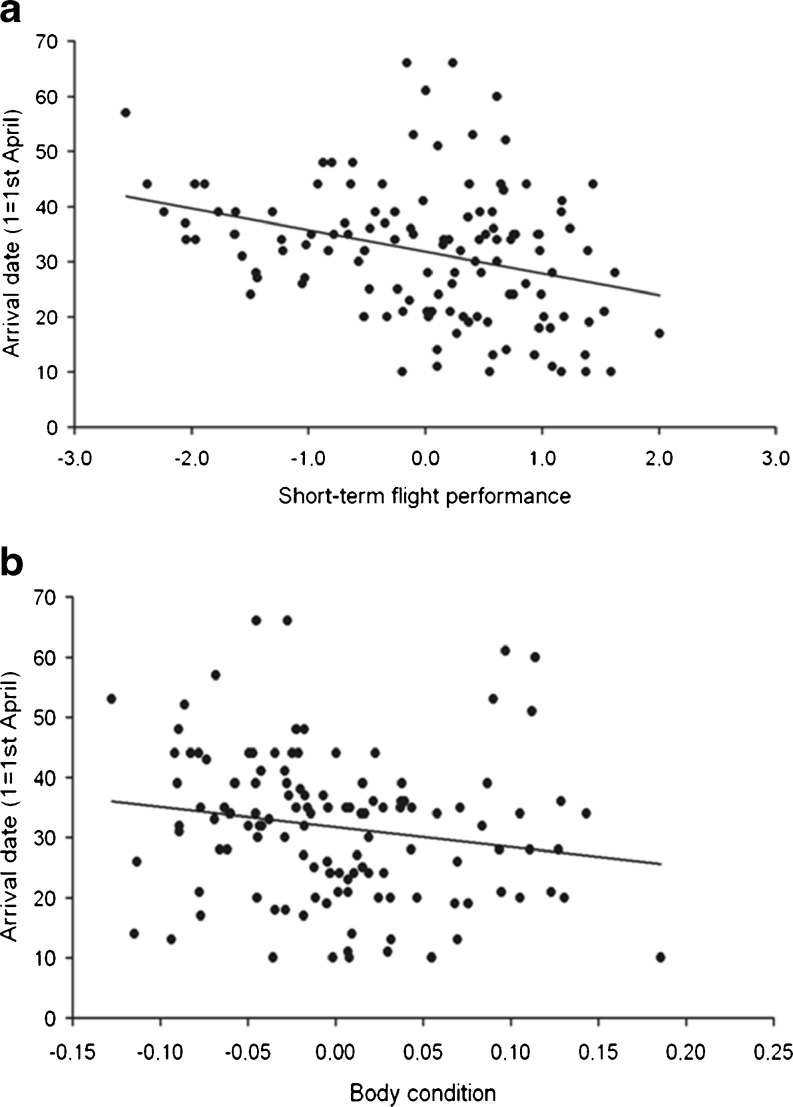
Spring arrival date in the barn swallow in relation to **a** short-term flight performance and **b** body condition. Flight performance is the first principal component extracted from birds’ flight manoeuvrability, maximum acceleration and maximum velocity as measured in flight tunnels. Condition is an index of body condition expressed as residuals from a linear regression of body mass measured shortly after return from spring migration against cubic tarsus length

Short-term flight performance of individual birds significantly predicted their spring arrival date in simple linear mixed effects model with sex, year and colony as additional predictors (Table 2). Specifically, earlier arrivals performed better in flight trials than later arrivals (Fig. 1a). Analogous analyses had a significant effect of body condition, tail length and age on the date of arrival (Table 2). Earlier arrivals were in better condition (Fig. 1b), had longer tails and were older than later arrivals.

**Table 2:** Simple mixed linear models estimating barn swallow spring arrival date with short-term flight performance, body condition, tail length and age, including sex, year of study and colony as additional predictors

Models (predictors)	Type III *F*	*DDF*	*P*
Flight performance	11.60	116.5	0.001
Sex	2.12	88.9	0.15
Year	0.02	42.8	0.88
Colony	0.03	96.9	0.86
Body condition	8.09	119.2	0.005
Sex	5.00	97.5	0.028
Year	0.42	44.1	0.52
Colony	0.62	97.2	0.43
Tail length	4.14	37.7	0.049
Sex	3.22	101.8	0.08
Year	0.01	39.9	0.91
Colony	0.84	97.9	0.36
Age	66.47	112.6	<0.001
Sex	4.15	101.4	0.044
Year	3.02	68.8	0.09
Colony	0.55	104.3	0.46

*DDF* denominator degrees of freedom (*N* = 124)

In my linear mixed effects model analyses including all predictor variables, the most likely models predicting spring arrival date were not strongly weighed (Table 1). Age and short-term flight performance received the highest summed Akaike weights, and thus the highest empirical support (Table 3). With a high cumulative AIC_c_ weight of 0.887, one can interpret short-term flight performance as having almost 90 % chance that it may indeed play a role in determining spring arrival order in the barn swallow. Body condition received smaller cumulative weight, with a low one for tail length. Analyses of overall model fit (Table 1) revealed that age was the most influential predictor variable, with short-term flight performance and body condition appearing to have considerable effects. The effect of tail length proved to be low. This was also supported because the 95 % confidence interval for flight performance, age and body condition did not include zero while that was not the case for tail length (Table 4).

**Table 3:** Akaike weights *ω*_*i*_ for the different predictor variables to explain spring arrival date in the barn swallow, summed over all models in which each variable features

Predictor variable	Summed *ω*_*i*_	Coefficients	SE
Age	1.000	−0.968	0.129
Flight performance	0.887	−0.181	0.066
Body condition	0.663	−0.102	0.053
Tail length	0.507	−0.053	0.035

Summed weights are used to estimate the relative importance of predictor variables (they express the probability that the variable affects the response variable). Model averaged coefficients (and standard errors) are also given as an indication of the effect size

**Table 4:** Results of the linear mixed effects model (the best model in Table 1) illustrating the effect of short-term flight performance, body condition, tail length and age on spring arrival date in the barn swallow (*N* = 124)

Predictor variable	Coefficients	SE	95 % CI	Type III *F*	*P*
Flight performance	−0.187	0.070	−0.326 to −0.047	7.030	0.009
Body condition	−0.158	0.072	−0.301 to −0.014	4.743	0.031
Tail length	−0.106	0.060	−0.227 to 0.015	3.057	0.085
Age	−0.949	0.128	−1.203 to −0.695	54.903	<0.001
Sex	0.481	0.153	0.177 to 0.785	9.833	0.002
Year	−0.270	0.115	−0.499 to −0.040	5.465	0.022
Intercept	0.434	0.134	0.169 to 0.699	0.859	0.356

## Discussion

Results obtained in this study can be summarised in two main findings. First, short-term flight performance was relatively strong and significant predictor of spring arrival date in the barn swallow. Birds that arrived earlier in spring were characterised by higher short-term flight performance than later arriving individuals (Fig. 1a). To my knowledge, this is the first study to show the existence of a relationship between locomotor performance and spring arrival date in a migratory animal. Second, short-term flight performance predicted arrival date independently of morphological variables—the degree of sexual ornamentation (the length of tail) and wing aspect ratio and body condition.

In this study, I focused on short-term flight performance—a composite variable that integrated three flight measures: manoeuvrability, acceleration and velocity. The three flight measures were significantly repeatable within season. Flight trials adopted in this study assumed that birds were escaping from flight tunnels. Moreover, the crowded stringed section of the flight maze forced the birds to make increasingly tight turns around obstacles. Flight performance displayed by the birds in such circumstances may be close to the maximum of manoeuvrability and acceleration (Warrick 1998; Bowlin and Winkler 2004; Rowe et al. 2001). Short-term flight performance may translate into migratory performance. Better fliers might be able to increase migration speed by reducing time spent foraging relative to time spent en route (Hedenström and Alerstam 1995; Alerstam and Hedenström 1998; Houston 2000). Migrating barn swallows may combine foraging with covering migration distance, and they may gather substantial fuel stores at stopovers prior to crossing wide geographical barriers (Rubolini et al. 2002; Turner 2006). In this species, higher short-term flight performance may translate into higher foraging success. Barn swallows prefer large, strong flying insects—typically large flies—which are more profitable to hunt as they provide higher net energy gain than smaller insects (Turner 1982; Brown and Brown 1999b). This preference is particularly strongly expressed during the nestling feeding stage of the breeding cycle, although barn swallows select such prey also during non-breeding season, provided active insects are available. Hunting active insects requires high manoeuvring and acceleration performance, and the barn swallow’s flight and bill morphology is well adapted to pursue and snatch such prey items (Waugh 1978; Thomas 1993). No study has investigated the relationship between flight performance and foraging success in the barn swallow. However, experimental manipulation of the flight morphology in this species suggests that flight performance and foraging success change in the same direction with progressive changes in the length of tail streamers (Møller et al. 1995; Evans 1998; Rowe et al. 2001; Bro-Jørgensen et al. 2007). Better fliers might also be prone to increase their airspeeds during migration more than the average individual; thus, short-term flight performance might affect directly the total duration of the migratory journey. Migrating birds are likely to fly on minimal time (*V*_mt_; or ‘full speed’), which is faster than the maximum range speed (*V*_mr_; that gives the longest distance on a given amount of fuel; Hedenström and Alerstam 1995). This can occur especially during the last stages of migration, when it is of prime importance for the bird to arrive at its destination before its competitors (Alerstam 2006; Karlsson et al. 2012).

Barn swallows are long-distance migrants, and the migratory round trip of the Polish population of this species annually covers >12,000 km between the breeding and wintering areas (Keith et al. 1992). I suggest that higher flight performance may help migrants to undergo such a long journey without compromising health and survival. In my study of annual survival in relation to short-term flight performance (P. Matyjasiak, unpublished data), barn swallow males that survived to the next breeding season were significantly better fliers than non-survivors (*t* test, *t* = −2.45, *df* = 64, *P* = 0.017; survivors, *n* = 30 and non-survivors, *n* = 36). No such difference was found among females (*t* test, *t* = −0.26, *df* = 56, *P* = 0.79; survivors, *n* = 28 and non-survivors, *n* = 30). This difference between the sexes can be explained by more intense competition for early arrival among male than female barn swallows (Møller 1994b). Females are not as constrained for early arrival as males, and hence they can afford to arrive later, thus avoiding the cost of hurried migration. If higher short-term flight performance improves the effectiveness of food acquisition in the barn swallow, it may help better fliers to acquire resources satisfying the demands of expenditure on migration more efficiently compared with the average bird. The pattern observed for arrival date on the one hand and annual survival on the other suggests that barn swallows can make use of their flight abilities to survive the long journey and to arrive early from Africa.

I have found that in the barn swallow, short-term flight performance predicted spring arrival time independently of condition and the size of the tail ornament. This finding is interesting for two reasons. First, it addresses the issue of the nature of the condition dependence of fitness-related traits. Condition is an important predictor of traits that are closely associated with fitness components (Andersson 1994; Tomkins et al. 2004). It reflects the capacity of individuals to acquire resources efficiently from the environment. If short-term flight performance is related to foraging success in the barn swallow, as discussed above, it represents a source of condition (Winkler and Allen 1995; Bowlin and Winkler 2004). For instance, it may assist in obtaining insect prey that provide valuable nutrients (e.g. cerotenoids and vitamins), which in turn play an important role in mediating the arrival timing (Ninni et al. 2004). In such a situation, it may mediate the condition dependence of spring arrival date: better fliers may be able to arrive at their breeding grounds earlier and in better body condition. Further research might investigate the direct and indirect effects of flight performance and condition on arrival date, using path analysis models. Second, it suggests that locomotor performance can be an index of individual quality in the context of spring arrival time that functions at least partially independently of the morphological quality indicator, which is the size of tail ornament. Higher short-term flight performance can assist male barn swallows in acquiring fitness benefits of early arrival. Simultaneously, better flyers can make valuable mates. Locomotor traits often are heritable (Garland et al. 1990; Le Galliard et al. 2004), and hence offspring may inherit high locomotory performance and associated advantages (resource acquisition ability and survival prospects) from their parents. Also direct benefits are possible, as partners who have higher flight performance may be more efficient at providing food for nestlings (Hakkarainen et al. 1996). It seems that short-term flight performance, itself being condition dependent, can accomplish a dual function in migrating birds: as a source of condition through its effect on foraging success and as an indicator of individual quality in the context of timing of arrival from migration. Future studies might investigate whether locomotor performance is a direct target of sexual selection in the context of the timing of spring arrival. It would address a currently important topic of whether and how locomotion leads to higher mating and reproductive success (Lailvaux and Irschick 2006; Husak and Fox 2008).

Summing up, the results of this study suggest that short-term flight performance is a significant and important predictor of timing of arrival from spring migration in a migratory bird. It predicted arrival time independently of the size of the tail ornament, which is an indicator of male quality in the barn swallow. This is the first empirical evidence, to my knowledge, of a relationship between flight performance and migratory performance. Future studies utilising the forthcoming innovative technologies for tracking migrant birds (e.g. Stutchbury et al. 2009; Bridge et al. 2011) are needed to investigate how birds actually migrate. It is unknown whether barn swallows differing in flight performance and condition winter in the same localities, or whether they exhibit synchrony in timing of migration. It might be examined whether better fliers are actually characterised by better migratory performance. Manipulative experiments are also necessary for better understanding causal relationships determining the condition-dependence of timing of arrival and the role of short-term flight performance in the context of arrival date.
